# Injurious mechanical ventilation affects neuronal activation in ventilated rats

**DOI:** 10.1186/cc10230

**Published:** 2011-05-13

**Authors:** María Elisa Quilez, Gemma Fuster, Jesús Villar, Carlos Flores, Octavi Martí-Sistac, Lluís Blanch, Josefina López-Aguilar

**Affiliations:** 1 CIBER de Enfermedades Respiratorias, Instituto de Salud Carlos III. C/ Sinesio Delgado 6, Madrid, 28029, Spain; 2 Critical Care Center, Corporació Sanitaria Parc Taulí, Institut Universitari, Esfera UAB. Parc Taulí sn. Sabadell, 08208, Spain; 3 Research Unit, Hospital Universitario Dr.Negrín. Barranco de la Ballena s/n. Las Palmas de Gran Canaria, 35010, Spain; 4 Research Unit, Hospital Universitario N.S. de Candelaria. Carretera del Rosario 145, Santa Cruz de Tenerife, 38010, Spain; 5 Universitat Autònoma de Barcelona. Campus de la UAB, Bellaterra, 08193, Spain

## Abstract

**Introduction:**

Survivors of critical illness often have significant long-term brain dysfunction, and routine clinical procedures like mechanical ventilation (MV) may affect long-term brain outcome. We aimed to investigate the effect of the increase of tidal volume (Vt) on brain activation in a rat model.

**Methods:**

Male Sprague Dawley rats were randomized to three groups: 1) Basal: anesthetized unventilated animals, 2) low Vt (LVt): MV for three hours with Vt 8 ml/kg and zero positive end-expiratory pressure (ZEEP), and 3) high Vt (HVt) MV for three hours with Vt 30 ml/kg and ZEEP. We measured lung mechanics, mean arterial pressure (MAP), arterial blood gases, and plasma and lung levels of cytokines. We used immunohistochemistry to examine c-fos as a marker of neuronal activation. An additional group of spontaneously breathing rats was added to discriminate the effect of surgical procedure and anesthesia in the brain.

**Results:**

After three hours on LVt, PaO_2 _decreased and PaCO_2 _increased significantly. MAP and compliance remained stable in MV groups. Systemic and pulmonary inflammation was higher in MV rats than in unventilated rats. Plasma TNFα was significantly higher in HVt than in LVt. Immunopositive cells to c-fos in the retrosplenial cortex and thalamus increased significantly in HVt rats but not in LVt or unventilated rats.

**Conclusions:**

MV promoted brain activation. The intensity of the response was higher in HVt animals, suggesting an iatrogenic effect of MV on the brain. These findings suggest that this novel cross-talking mechanism between the lung and the brain should be explored in patients undergoing MV.

## Introduction

Acute lung injury (ALI) and the acute respiratory distress syndrome (ARDS) are associated with high morbidity and mortality [1], and ARDS survivors present significant long-term cognitive impairment [2]. These consequences may result from complex interactions between different clinical protocols and endogenous factors occurring simultaneously in critically ill patients [3]. In this context, mechanical ventilation (MV) is a lifesaving procedure but not without complications. Even in healthy lungs, MV may contribute to a positive feedback loop that starts with mechanotransduction (in lungs) at the epithelial and endothelial levels leading to a deleterious inflammatory cascade that might affect distant organs and systems [4-6]. Moreover, critical care patients who undergo long-term MV show distinctive neurological impairment, including memory and cognitive decline [7].

Many studies have examined the mechanisms involved in the neuroimmune crosstalk; most focus on the central nervous system (CNS) response to systemic inflammation. However, the mechanisms through which damage to remote organs can reach the brain are poorly understood [8,9], including early neurological effects related to MV and the importance of settings used.

The immediate early gene (IEG) c-fos has been used as a marker of neuronal activity, and correlates with an increase in electrical and metabolic activity in brain cells by pathological situations, also involved in phenomena of neuronal plasticity, amongst others. C-fos is expressed in response to a wide range of stimuli and is implicated in processes such as transcription of genes, apoptosis or proliferation [10]. In order to make a first approximation to the crosstalk between brain-lung during MV, we have used this rapidly induced IEG, according to the time course of the study (three hours) as a tool to elucidate early neurological changes that might be associated with lung injury.

The main objective of the present study was to investigate the effect of the increase in tidal volume on activation in some areas of the brain in a rat model of MV, using c-fos. Therefore, we compared rats ventilated with two different injurious ventilatory strategies, a high Vt group *vs*. a low Vt group and vs. spontaneously breathing or basal rats.

## Materials and methods

### Animal preparation

This study was approved by the Animal Ethics Committee at the Corporació Sanitaria Parc Taulí, Barcelona, Spain. We studied 24 adult male Sprague-Dawley rats weighing 350 to 370 grams housed in standard conditions with food and water *ad libitum*. Animals were anesthetized with intraperitonial ketamine (75 mg/kg) and xylazine (10 ml/kg), tracheotomized, and paralyzed with intravenous succinylcholine (2 ml/kg). An endotracheal tube (2 mm inner diameter) was inserted and tightly tied to avoid air leaks, and rats were ventilated using a Servo 300 ventilator (Siemens, Solna, Sweden). Vt was set and measured using the ventilator's pneumotachograph. Airway pressure was monitored via a side port in the tracheal tube using a pressure transducer (Valydine MP45, Valydine Engineering, Northridge, CA, USA). The left carotid artery was cannulated and connected to a pressure transducer (Transpac Monitoring Kit; Abbot, Sligo, Ireland) to monitor mean arterial pressure (MAP). The right jugular vein was cannulated for fluid infusion. Blood and airway pressures were routed to an amplifier (Presograph, Gould Godart, Netherlands), converted to digital (Urelab, Barcelona, Spain), and recorded in a personal computer (Anadat-Labdat Software, RTH InfoDat, Montreal, QC, Canada). Then, animals were randomly assigned to one of three experimental groups (*n *= 8 in each group): (i) Basal group (BAS), unventilated animals, were immediately exanguinated after induction of the anesthesia (ii) Low Vt group (LVt), ventilated with 8 ml/kg and a positive end-expiratory pressure (PEEP) of 0 cmH_2_O (ZEEP) for three hours, and (iii) High Vt group (HVt), ventilated with 30 ml/kg and ZEEP for three hours. To maintain normocapnia without decreasing respiratory rate, instrumental dead space was increased in the HVt group. An additional group of spontaneously breathing rats (Spont) was added to discriminate the effect of the surgical procedure and anesthesia on brain activation. The spontaneously breathing animals were anaesthesized and the same surgical procedure as ventilated groups (cannulation and tracheostomy) was performed. No mechanical ventilation was applied in this group. Identical patterns of fluid infusion and anaesthesia were applied in the three groups maintained in protocol during three hours (spont, LVt, HVt).

### Experimental protocol

At baseline, animals in the MV groups underwent volume-controlled ventilation with 8 ml/kg Vt and 2 cmH_2_O PEEP. Inspired oxygen fraction (FiO_2_) was kept at 0.4 throughout the experiment, and the respiratory rate was adjusted for normocapnia. We measured values of MAP, arterial blood gases, and respiratory system parameters 15 minutes after initiating MV (baseline) and hourly thereafter after randomization. Inspiratory and expiratory pauses were applied to calculate static lung compliance (Crs). Fluid management was identical in all groups (Ringer-lactate, 10 mL/kg/h) to prevent differences that might favour edema formation and vasoactive drugs were not used in any group. At the end of the three-hour period, rats were euthanatized by exsanguination. We centrifuged 7 ml of blood from each animal and stored the plasma at -80°C for protein determinations. Hearts and lungs were removed *en bloc*, and the right lung was frozen for additional tissue analyses of proteins. Rats' brains were removed from the cranium by careful dissection and immediately frozen and stored at -80°C.

### Histological analysis

Left lungs were fixed by instillation of 4% buffered formaldehyde into the airway at a pressure of 5 cmH_2_O and immersed in the same fixative. Two investigators blinded to experimental groups calculated histological scores after hematoxylin-eosin (HE) staining as described elsewhere [11] and assessed intra-alveolar neutrophil infiltration by counting the number of neutrophils in fifty fields per animal at a magnification of X400 using ImageJ v1.40g (Wayne Rasband, NIH, USA).

Lung damage was determined using a Lung Injury Score (LIS), based on the evaluation of alveolar edema, hemorrahage, neutrophil infiltration and alveolar septal thickening in each animal. Each parameter was scored from 0 to 4. Subsequently, the total LIS was calculated by adding the indicidual score for each parameter, up to a maximum score of 16 [12].

### Plasma and lung protein immunoassays

Commercially available enzyme-linked immunosorbent assay (ELISA) kits (Biosource, Camarillo, CA, USA) were used to determine the following plasma/lung protein levels: tumor necrosis factor-alpha (TNF)-α, macrophage inflammatory protein (MIP-2), interleukin (IL)-6, IL-1β, monocyte chemoattractant protein (MCP-1), and IL-10. Analyses of all samples, standards, and controls were run in duplicate following the manufacturer's recommendations.

### Brain immunohistochemistry

The brain areas of interest were cut into 20-μm coronal sections (CM1900, Leica Microsystems, Wetzlar, Germany) and stored at -80°C. Sections were processed for single immunohistochemistry using a rabbit polyclonal antibody against c-fos (c-fos (4), Santa Cruz Biotechnology Inc., Santa Cruz, CA, USA) diluted 1:250 The immunoreaction was visualized with diaminobenzidine and H_2_O_2 _[13]. Additional sections were stained with cresyl violet to identify the regions of interest: thalamus, retrosplenial cortex (RS), central amygdala (CeA), hippocampus, paraventricular hypothalamic nuclei (PVN), and supraoptic nucleus (SON). After immunostaining, specific activated areas were identified by light microscopy (DM250, Leica, Wetzlar, Germany) with the aid of a stereotaxic atlas [14]. Brain sections were digitized and c-fos-positive cells were evaluated according to the intensity of staining and then semiquantified using Image J software (ImageJ 1.40g, W. Rasband, NIH, USA). An optimal threshold was set for all sections to minimize background signals.

### Statistical analysis

We used power analysis for ANOVA designs to estimate the sample size assuming an α error of 0.05 and β error of 0.2 (Granmo 5.2 software, Barcelona, Spain). All values are expressed as mean ± SEM. U-Mann-Withney non-parametric tests were used to analyze differences between groups, under the supervision of an expert statistician (SPSS 17.0 software, Chicago, IL, USA). Significance was set at *P *< 0.05.

## Results

Animal body weights were similar in all groups. At baseline, no differences in hemodynamics or gas exchange were observed between MV groups. Basal rats were exsanguinated at time zero and were used as the baseline group in comparisons between groups. No animals died during the experimental period.

### Physiological variables

**Figure 1:**
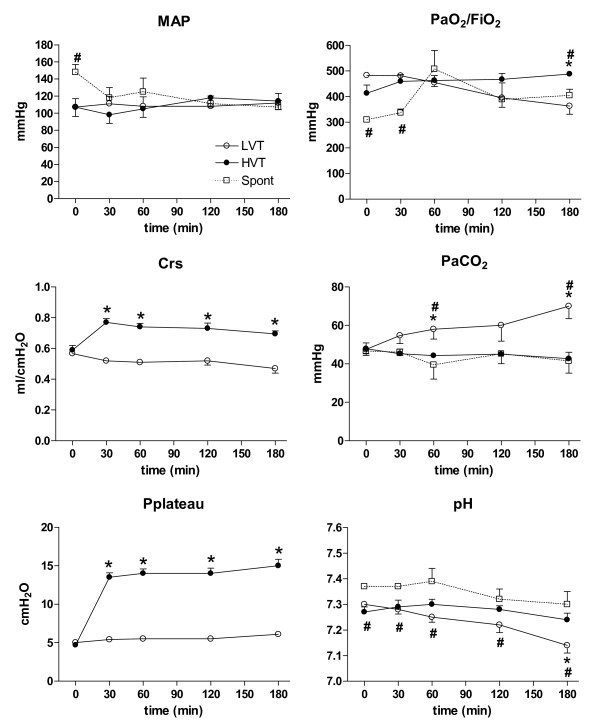
**Hemodynamic and respiratory characteristics of rats during the three-hour period**. No differences between groups were observed at baseline. MAP remained stable in both groups. Pplateau and Crs increased significantly during HVt ventilation but remained stable during the three-hour period. There were no differences between LVt and HVt in Pa/FiO_2_. pH in animals spontaneously breathing was slightly higher than in animals receiving MV. PCO2 increased only in LVt animals. Data are presented as mean ± SE. *: *P  *< 0.05 versus the HVt group, and #: *P  *< 0.05 vs Spont group. N = 8 animals per group. Abbreviations: MAP, mean arterial pressure; BAS, basal; LVt, low tidal volume; HVt, high tidal volume; Spont, spontaneous breathing; Pplateau, plateau pressure; Crs, static compliance of the respiratory system.

MAP remained stable within the normal range throughout the three-hour period in all groups (Figure 1). Respiratory system compliance (Crs) and plateau pressure (Pplateau) increased with HVt MV, but both remained unchanged throughout the experimental period (Figure 1). Respiratory rates were not significantly different between LVt and HVt animals (mean 47.3 *vs *47, respectively; *P *= 0.7). Significant decreases in PaO_2_/FiO_2 _and pH and concurrent increases in PaCO_2 _were found in LVt animals after three hours of MV (Figure 1). pH in animals spontaneously breathing was slightly higher than in those receiving MV, and remained stable during the experiment.

### Histology

**Figure 2:**
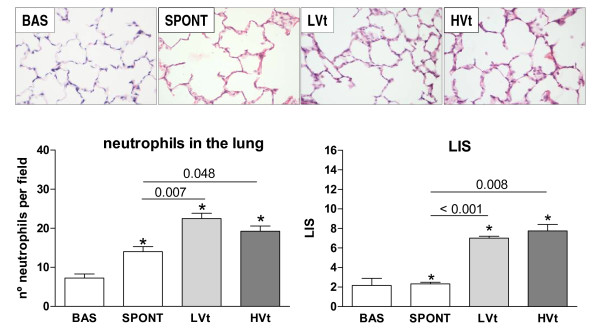
**Representative images of lungs in each group after H-E staining and LIS**. The percent of lung neutrophil content and LIS increased with MV but was similar in animals receiving LVt and HVt. Results are represented as mean ± SE. *P  < 0.05 versus the unventilated basal group. N = 8 animals per group. Abbreviations: BAS, basal; LVt, low tidal volume; HVt, high tidal volume; Spont, spontaneous breathing; LIS, Lung injury score.

Figure 2 shows representative images of lungs in each experimental group. Lung neutrophilic infiltration and LIS were significantly higher in MV rats than in unventilated rats, but no differences between LVt and HVt were found.

### c-Fos immunopositive brain areas

**Figure 3:**
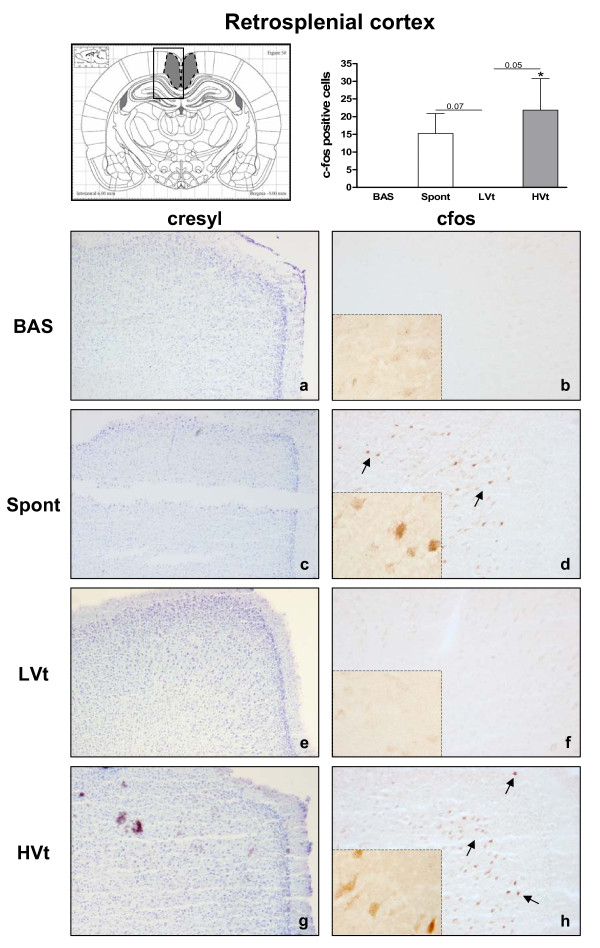
**Brain activation evidenced by c-fos immunotreactivity in the retrosplenial cortex**. On the top (left): Coronal section diagram encompassing the area of interest. On the bottom: Representative images of RS from each experimental group after cresyl violet staining (left, **a,c,e,g**, 40X) and c-fos immunohistochemistry (right 100X and 400X). Brown dots represent c-fos staining. Black arrows indicate some examples of c-fos positive cells. HVt (h) and spontaneous breathing (d) increased the number of c-fos-positive neurons in the RS; lower levels of c-fos immunoreactive cells were found in unventilated (b) and LVt (f) animals. Data are presented as mean ± SE. *P  < 0.05 respect to unventilated basal animals. Abbreviations: BAS, basal; LVt, low tidal volume; HVt, high tidal volume; Spont, spontaneous breathing; RS, retrosplenial cortex.

**Figure 4:**
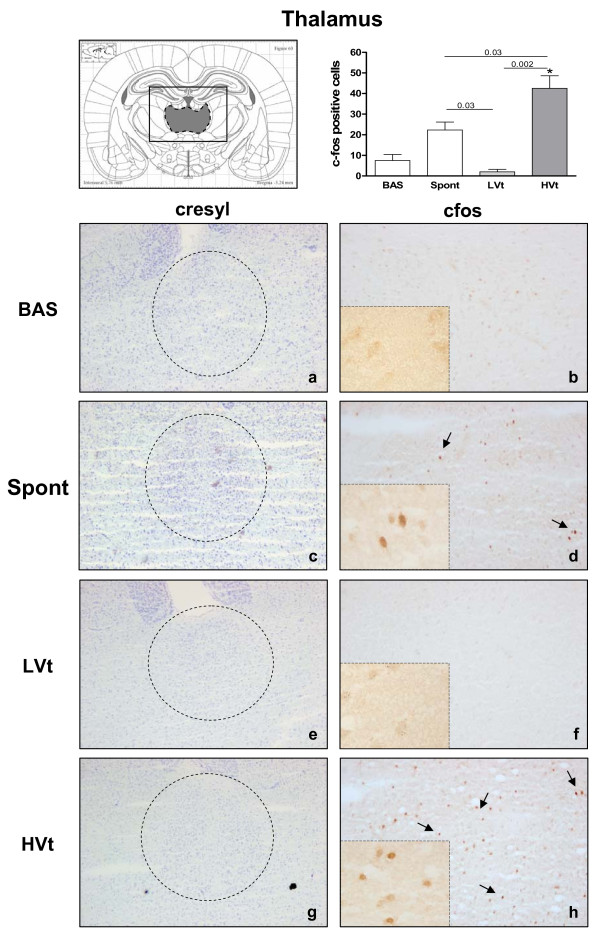
**Brain activation evidenced by c-fos immunotreactivity in thalamus**. On the top (left): Coronal section diagram encompassing the area of interest. On the bottom: Representative images of thalamus from each experimental group after cresyl violet staining (left, **a,c,e,g**, 40X) and c-fos immunohistochemistry (right 100X and 400X). Brown dots represent c-fos staining. Black arrows indicate some examples of c-fos positive cells. HVt (h) and spontaneous breathing (d) increased the number of c-fos-positive neurons in the thalamus. Data are presented as mean ± SE. *P  < 0.05 respect to unventilated basal animals. Abbreviations: BAS, basal; LVt, low tidal volume; HVt, high tidal volume; Spont, spontaneous breathing.

**Figure 5:**
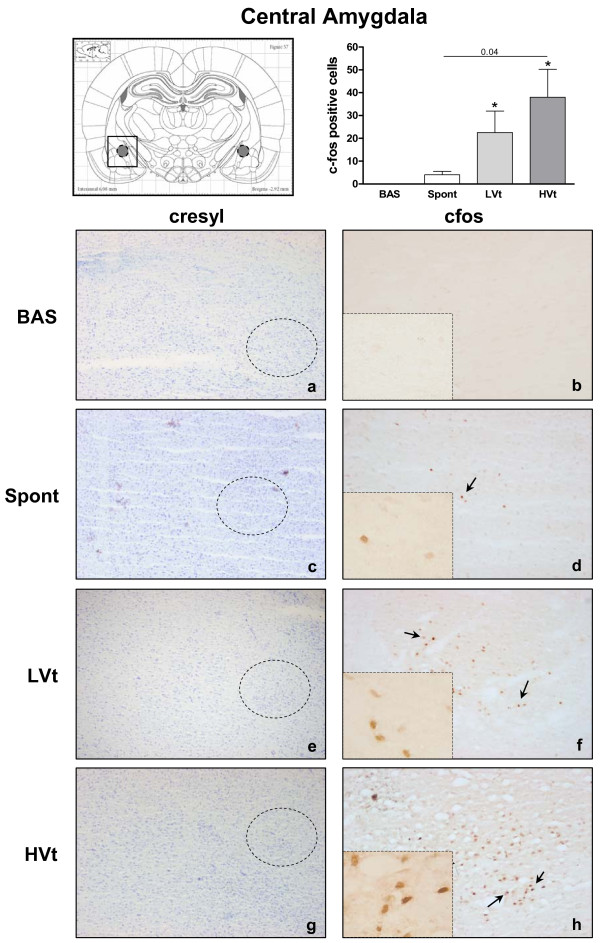
**Brain activation evidenced by c-fos immunotreactivity in central amygdala**. On the top (left): Coronal section diagram encompassing the area of interest. On the bottom: Representative images of the central amygdala from each experimental group after cresyl violet staining (left,**a, c,e,g**, 40X) and c-fos immunohistochemistry (right, 100X and 400X). Brown dots represent c-fos staining. Black arrows indicate some examples of c-fos positive cells. MV significantly increased c-fos immunoreactive cells in the CeA independently of the Vt level (**f, h **). Few c-fos positive cells were found in CeA of spontaneous breathing (**d**) and basal (**b**) animals. Data are presented as mean ± SE. *P  < 0.05 respect to unventilated basal animals. Abbreviations: No MV, unventilated animals; LVt, low tidal volume; HVt, high tidal volume; Spont, spontaneous breathing; CeA, central amygdala.

**Figure 6:**
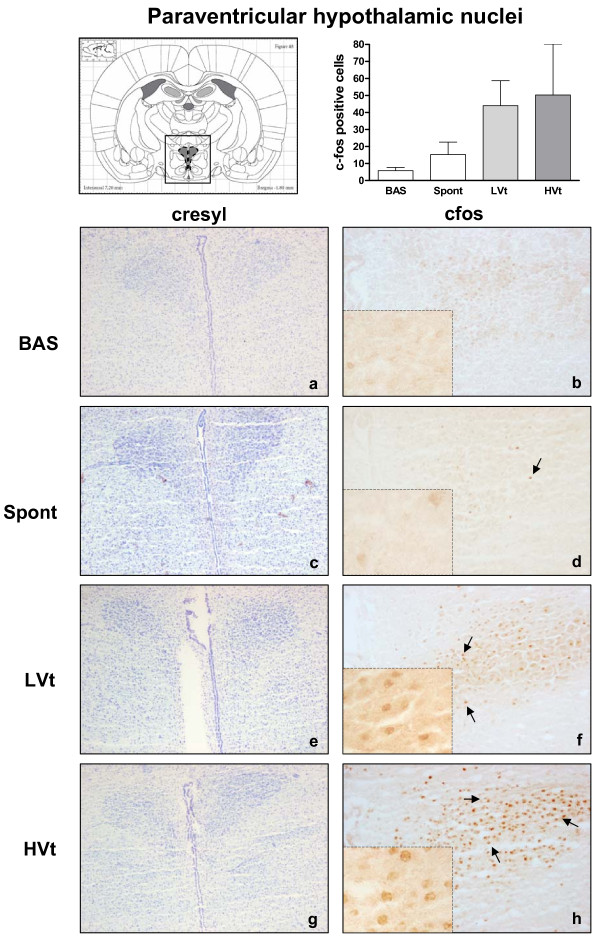
**Brain activation evidenced by c-fos immunotreactivity in Paraventricular hypothalamic nuclei**. On the top (left): Coronal section diagram encompassing the area of interest. On the bottom: Representative images of Paraventricular hypothalamic nuclei from each experimental group after cresyl violet staining (left, **a,c,e,g**, 40X) and c-fos immunohistochemistry (right 100X and 400X). Brown dots represent c-fos staining. Black arrows indicate some examples of c-fos positive cells c-fos expression in the PVN tended to increase with MV (**f, h**), but this increase did not reach significance compared with basal (**b**) or spontaneous breathing rats (**d**). Data are presented as mean ± SE. *P  < 0.05 respect to unventilated basal animals. Abbreviations: BAS, basal; LVt, low tidal volume; HVt, high tidal volume; Spont, spontaneous breathing; PVN, Paraventricular hypothalamic nuclei.

Neuronal activation evidenced by an increased number of c-fos immunopositive cells was observed in the RS (Figure 3) and thalamus (Figure 4) of HVt rats, but not in LVt or basal rats. c-fos expression was also observed in the CeA (Figure 5), PVN (Figure 6), and SON (data not shown) of MV rats, although activation did not differ between HVt and LVt animals. Similarly, no differences in c-fos activation in other cortical areas or in the hippocampus were observed between the experimental groups (data not shown).

Animals breathing spontaneously showed similar levels of activation in CeA and PVN than those observed in the basal group (Figures 5 and 6). Conversely, the c-fos signal in RS and Thalamus was higher than those found in BAS and LVt groups (Figures 3 and 4).

### Inflammatory mediators: plasma and lung protein levels

**Figure 7:**
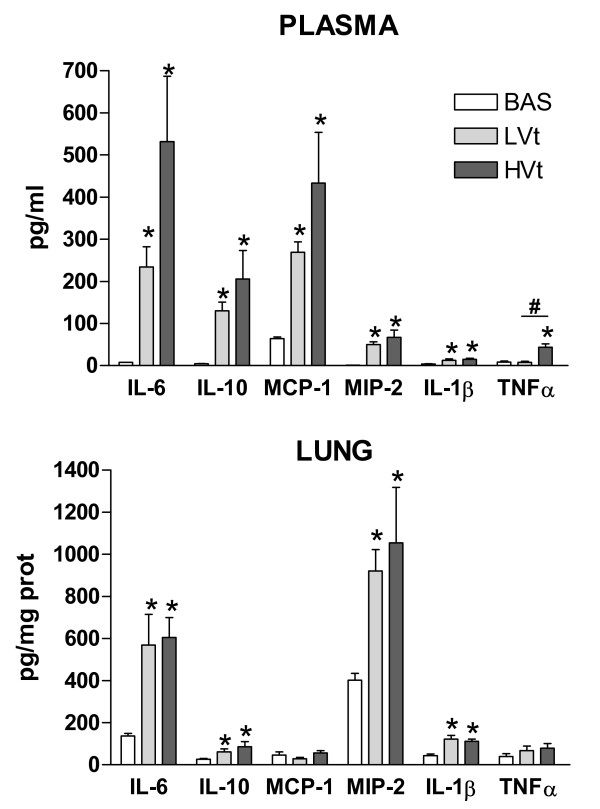
**Plasma and lung levels of proteins involved in the inflammatory cascade**. Mechanical ventilation triggered lung and systemic inflammatory responses. Compared to LVt, HVt promoted an increase in inflammatory markers mainly mediated by TNFα at the plasma leve. Data are presented as mean ± SE. *P  < 0.05 respect to unventilated basal animals, # *P  *< 0.05 vs LVt. *n *= 8 animals per group. Abbreviations: BAS, basal; LVt, low tidal volume; HVt, high tidal volume; Spont, spontaneous breathing; IL, interleukin, TNF, tumor necrosis factor; MCP, monocyte chemotactic protein; MIP, macrophage-inflammatory protein.

Figure 7 shows plasma and lung levels of pro- and anti-inflammatory mediators. MV increased plasma levels of IL-6, IL-10, IL-1β, MCP-1, and MIP-2, irrespective of the Vt level (LVt or HVt) (*P  *< 0.05). However, plasma TNFα levels increased significantly after three hours of HVt ventilation (*P *= 0.005) but remained unaltered in the LVt group.

In the lungs, irrespective of the Vt level, MV increased IL-6, IL-10, IL-1β, and MIP-2 levels (Figure 7). Lung TNFα levels were similar in MV and unventilated animals. We also observed a trend to higher MCP-1 in HVt compared to LVt (Figure 7). Taken all together, the inflammatory response was higher (but also more variable) in the HVt group than in the LVt group.

## Discussion

We found that MV induced c-fos expression in discrete areas of the brain in healthy and non-hypoxemic rats. Moreover, HVt ventilation caused more c-fos expression when compared to LVt ventilation, thus supporting the hypothesis that an iatrogeneous effect of MV may affect the brain. These results provide novel and important data that might have clinical relevance during the management of critically ill patients.

The immediate early gene *c-fos *[15] is rapidly induced and can be detected by immunochemistry; therefore it is a valuable tool for determining which brain areas are stimulated [16,17]. The basal expression of c-fos is low, but can be dramatically induced by a variety of stimuli and conditions such as metabolic stress, ischemia, and inflammation, among others [18,19]. Various mechanisms are probably involved in the response to MV. Lungs can "sense" mechanical stimuli by lung mechanoreceptors that can communicate to the brain. The autonomic nervous system is also involved in this crosstalk [20-22].

The ventilatory strategy may also affect the CNS by altering the inflammatory response at the lung level. In the present study, as reported elsewhere, we have used two different injurious MV strategies that triggered proinflammatory responses even in subjects receiving LVt [4,5,23]. The proinflammatory response to HVt was found mainly at the systemic level and was mediated by TNFα [6,23,24]. Only minimal differences in other cytokines, lung function parameters or LIS were found between MV groups. The release of inflammatory mediators [23,24] or certain metabolites to the bloodstream can be sensed by the brain, altering the permeability of the blood brain barrier [22,25] or modifying cerebral blood flow. No data are available about the contribution of these two mechanisms in the activation observed in the brain areas studied in our model.

We focused our study on brain areas involved in body homeostasis [26] and related to the Hypothalamic-Pituitary-Adrenal (HPA) axis, a major part of the neuroendocrine system that controls reactions to stress and regulates many body processes. In our study, the CeA, SON, and PVN were c-fos immunopositive after three hours of injurious MV but not in BAS or spontaneously breathing animals [25,27].

HVt consistently increased c-fos in the RS and thalamus, neither of which were activated in LVt or BAS animals. Interestingly, these two areas have also been activated in the spontaneously breathing animals. These results do not allow us to discriminate the role played by anesthesia and surgical procedures, since activation is minimal in LVT animals, which have been also submitted to the same experimental protocol. All these data suggest that the mechanisms inducing cell activation in these brain areas are different in HVT and spontaneously breathing animals. Moreover, in the literature, RS and thalamus have been linked to neurological disorders after stress [28,29], fatigue-loading in rats [30,31]; emotional or psychological stress might also induce neuronal activation in cortical and limbic regions [16,32].

In the present study we cannot determine whether the regional brain activation observed in LVt group was caused by moderate hypercapnia. This impaired gas exchange in the LVt group is compatible with progressive alveolar de-recruitment in the absence of PEEP. The higher level of brain activation observed in the HVt group occurred in the context of normocapnia, thus suggesting that mechanically-induced stress in the lung could promote c-fos activation in certain brain areas through other mechanisms, which deserves being explored in further investigations.

We found no differences between groups in the activation of the hippocampus (data not shown), which plays a role in the negative inhibition of the HPA stress axis through the abundant expression of glucocorticoid receptors [33] and is considered a potential target for sepsis treatment [8,34].

Our results were obtained in the context of preserved lung function and hemodynamic stability. The magnitude of the response to HVt observed by different authors varies [23,24,35-37], and some authors have reported detrimental effects of HVt on MAP [38]. However, we found that adequate fluid management ensured MAP stability throughout the experimental procedure (three hours), corroborating our previous findings [11,23]. Therefore, the differences in the results could not be attributed to differential organ perfusion.

### Limitations of the study

Animal models of complex diseases are potentially limited by interspecies differences and have no immediate applicability to humans; nevertheless, animal models are accepted as a valid strategy for the initial approach to multifactorial conditions. In this sense the HVt level applied in this work is not clinically relevant, but has been used to simulate those areas exposed to locally high pressures in injured lungs. Nevertheless, HVt application in our model of healthy lungs resulted only in a moderated increase in Pplat, compared with other more aggressive models in the literature [39,40]. Whereas this approach did preclude functional lung alterations, the short duration of MV also limited the analysis of brain alterations to early events like neuronal activation detected by c-fos immunostaining. Furthermore, our study design does not allow us to conclude whether inflammation and c-fos increased expression are mechanistically linked, let alone the nature of this possible link. In fact, as mentioned, hypercapnia would contribute to brain activation by a different mechanism. Moreover, the tight control of anesthesia and neuromuscular paralysis used in MV groups precluded any differences in this regard. Spontaneously breathing animals served to explore the effect of instrumentation and anesthesia, as they were not paralyzed.

### Clinical relevance

Due to the novelty of this issue (brain activation and MV) and the limitations of the study, we can only speculate about the translation of these results to the clinical setting. The etiology of cognitive impairment in critically ill patients is undoubtedly multifactorial and is the subject of ongoing discussion [2,3]. Nevertheless, crosstalk between the lung and brain is poorly understood [22], and although many randomized controlled clinical trials have evaluated the efficacy and safety of various methods of MV in ARDS and ALI patients, few studies have explored the influence of MV patterns at the neuronal level. Our findings about regional brain activation during MV could help define particular areas susceptible to be activated by mechanoreceptors in the lung. Those areas might play a crucial role in regulating early events occurring during the application of non-adequate MV patterns. Our findings might have implications for understanding how the brain senses incoming signals or insults from the lungs in anesthetized and paralyzed subjects.

## Conclusions

Our data further support the concept of brain-lung interaction during MV and indicate the importance of the ventilatory settings used. These findings may, therefore, have clinical relevance and emphasize the importance of further research in this field.

## Key messages

• Injurious mechanical ventilation might be associated with neuronal activation in discrete areas of the brain

• A high tidal volume might play a synergistic role on c-fos expression in some areas of the brain.

• The release of inflammatory mediators to the bloodstream could be involved in the lung-to-brain interaction during mechanical ventilation.

• Lung-brain cross-talking is an emerging area of research in critically ill patients receiving mechanical ventilation.

## Abbreviations

ALI: Acute Lung Injury; ARDS: Acute Respiratory Distress Syndrome; BAS: Basal; CeA: Central amygdale; CNS: Central Nervous System; Crs: Compliance of the respiratory system; ELISA: Enzyme-Linked immunoabsorbent assay; FiO_2_: Inspired oxygen fraction; HE: Hematoxilin-Eosin; HPA axis: Hypothalamic-pituitary-adrenal axis; HVt: High Tidal volume; IEG: immediate early gene; IL: Interleukin; LIS: Lung injury score; LVt: Low Tidal volume; MAP: Mean arterial pressure; MCP-1: Monocyte chemoattractant protein-1; MIP-2: Macrophage inflammatory protein 2; MV: Mechanical ventilation; NTS: Tractus solitarius nucleus; PEEP: End expiratory pressure; Pplateau: Plateau pressure; PVN: Paraventricular nucleus; RS: retrosplenial cortex; SON: Supraoptic nucleus; Spont: Spontaneous breathing; TNF: Tumor necrosis factor; Vt: tidal volume; ZEEP: Zero end expiratory pressure.

## Competing interests

The authors declare that they have no competing interests.

## Authors' contributions

MEQ, OMS, JLA and LLB were responsible for the original design. MEQ, GF and JLA were responsible of experimental procedure and analysis. JLA and OMS were responsible for data management. MEQ carried out the statistical analysis and wrote the initial draft supervised by JLA and LLB. JV, CF and OMS critically revised the manuscript. All authors contributed and approved the final version of the manuscript.
