# Erratum to: Injurious mechanical ventilation affects neuronal activation in ventilated rats

**DOI:** 10.1186/s13054-015-1076-5

**Published:** 2015-10-27

**Authors:** María Elisa Quilez, Gemma Fuster, Jesús Villar, Carlos Flores, Octavi Martí-Sistac, Lluís Blanch, Josefina López-Aguilar

**Affiliations:** CIBER de Enfermedades Respiratorias, Instituto de Salud Carlos III, C/ Sinesio Delgado 6, Madrid, 28029 Spain; Critical Care Center, Corporació Sanitaria Parc Taulí, Institut Universitari, Esfera UAB. Parc Taulí sn, Sabadell, 08208 Spain; Research Unit, Hospital Universitario Dr.Negrín, Barranco de la Ballena s/n, Las Palmas de Gran Canaria, 35010 Spain; Research Unit, Hospital Universitario N.S. de Candelaria, Carretera del Rosario 145, Santa Cruz de Tenerife, 38010 Spain; Universitat Autònoma de Barcelona, Campus de la UAB, Bellaterra, 08193 Spain

Unfortunately, the original version of this article [1] contained an error. Affiliation no. 5 had been omitted to be included for the first author, María Elisa Quilez. This has now been corrected. The full list of affiliations for the first author are:

María Elisa Quilez^1,2,5^

1 CIBER de Enfermedades Respiratorias, Instituto de Salud Carlos III. C/ Sinesio Delgado 6, Madrid, 28029, Spain

2 Critical Care Center, Corporació Sanitaria Parc Taulí, Institut Universitari, Esfera UAB. Parc Taulí sn. Sabadell, 08208, Spain

5 Universitat Autònoma de Barcelona. Campus de la UAB, Bellaterra, 08193, Spain
